# Aqueduct-fourth ventricle-cisterna magna shunting as a prophylactic procedure for postoperative hydrocephalus in selective fourth ventricular tumors: A retrospective study

**DOI:** 10.1097/MD.0000000000043697

**Published:** 2025-08-22

**Authors:** Min Tang, Seidu A. Richard, Yanming Ren, Bowen Huang, Zhigang Lan, Yuekang Zhang

**Affiliations:** a Department of Neurosurgery, West China Hospital, Sichuan University, Chengdu, Sichuan Province, P. R. China; b The Anesthesia and Surgery Center, Cheng Du Shang Jin Nan Fu Hospital, Sichuan University, Chengdu, Sichuan Province, P. R. China; c Institute of Neuroscience, Third Affiliated Hospital, Zhengzhou University, Zhengzhou, Henan Province, China; d Department of Biochemistry and Forensic Sciences, School of Chemical and Biochemical Sciences, C. K. Tedam University of Technology and Applied Sciences (CKT-UTAS), Navrongo, Ghana; e Department of Neurosurgery, West China Xiamen Hospital, Sichuan University, Xiamen, Sichuan Province, P. R. China.

**Keywords:** AFVCM shunting, FVTs, hydrocephalus, postoperative, torkildsen, ventriculostomy

## Abstract

Surgery for fourth ventricular tumors (FVT) is plagued by potential CSF blockage after the tumor removal due to a plethora of reasons. Thus, we explored the use of aqueduct-fourth ventricle-cisterna magna (AFVCM) shunting as prophylactic procedure for postoperative hydrocephalus after FVT resection. We retrospectively gathered the data of patients who underwent surgery of the fourth ventricle tumor between January 2019 and December 2021 at the Department of Neurosurgery in West China Hospital of Sichuan University. In all, a total of 96 selective cases were selected among 237 fourth ventricle tumor cases because of their superior tumor extension into the aqueduct. The patients were categorized into AFVCM shunting and no AFVCM shunting groups and risks factors for the development of postoperative hydrocephalus evaluated and their clinical outcomes were compared. In all 20 patients in the no AFVCM shunting group developed postoperative hydrocephalus out of the 96 patients included in the study. Univariate analysis revealed that AFVCM shunting (*P* = .0062) salvaged postoperative hydrocephalus. Multivariate analysis revealed that type of tumor (*P* = .017; OR = 1.076; 95% CI = 0.814–1.422), tumor size (*P* = .025; OR = 1.057; 95% CI = 0.516–2.165), residual tumor (*P* = .005; OR = 1.466; 95% CI = 0.833–2.581) were factors associated with postoperative hydrocephalus. However, AFVCM shunting (*P* = .029; OR = 5.47265E−13; 95% CI = 0.000–inf) salvaged postoperative hydrocephalus. AFVCM shunting may prevent postoperative hydrocephalus and reduce costs in patients with superior tumor extension into the aqueduct and failed total tumor resection.

## 1. Introduction

Fourth ventricle tumors (FVTs) are characteristically associated with high rate of postoperative hydrocephalus due to the compression of the tumor on the fourth ventricle, a key cerebrospinal fluid (CSF) pathways and other vital structures around it.^[1–4]^ The pathophysiology of FVT is depicted with stenosis triggered by inflammatory process as well as scarring involving the ependymal lining at the cerebral aqueduct and CSF pathways such as the ventricles.^[5–7]^ Anatomically, the roof as well as walls of the fourth ventricle contain vital structures of the efferent cerebellar pathway such as the dentate nuclei and the superior cerebellar peduncle whereas the floor of the fourth ventricle, formed by the brainstem, contains numerous cranial nerve nuclei on its surface.^[8–10]^

Notably, although tumor resection often resolves CSF circulation, 10% to 30% of patients may present with persistent hydrocephalus after removal of posterior fossa tumors which often borders the fourth ventricles.^[11–13]^ Thus, surgeries for FVTs are depicted with potential complications due to proximity to arcane eloquent structures as well as the risk of injury to perforating arteries supplying subcortical regions.^[4,9,14]^ Pre- and postoperative hydrocephalus often present with signs and symptoms of increased intracranial pressure.^[4,15–17]^

Interestingly, there are inconsistent results on the relationship between preoperative hydrocephalus as well as postoperative hydrocephalus and there is still no harmony in the management of hydrocephalus before and after FVT surgeries.^[4]^ Currently, shunting procedure such ventriculoperitoneal (VP) and endoscopic third ventriculostomy (ETV) are the widely use treatment modalities for the management of hydrocephalus in patients with FVTs.^[3,4,18–23]^ ETV appear to be more safe and durable means of controlling obstruction hydrocephalus compared to shunting.^[19,20,22]^ Although both modalities are associated with failures, the risk of ETV failure may be lower than the risk of VP shunt failure surgery.^[19,20,22]^

Herein, we used aqueduct-fourth ventricle-cisterna magna (AFVCM) shunting as a prophylactic for postoperative hydrocephalus after FVT removal by placing a catheter from the aqueduct through fourth ventricle towards the cisterna magna. Our AFVCM shunting is similar to Torkildsen shunt because in both procedures CSF drainage if confined inside the skull. However, insertion of shunts in our study are quite different from Torkildsen shunting. The Torkildsen shunt was first successfully performed by Dr Arne Torkildsen, a Norwegian neurosurgeon in 1937, and described in1939.^[24–28]^ It is an internal ventriculocisternal shunt that diverts the CSF flow from one of the lateral ventricles, specifically the occipital horn, to the cisterna magna of the posterior fossa.^[25,26]^ This procedure was widely accepted as an efficient surgical procedure for the management of hydrocephalus until the introduction of the ventriculoatrial shunts and later of the VP shunt procedures, when it was essentially abandoned.^[25,26]^

Notably, postoperative hydrocephalus which is a major and challenging complication after tumor resection in FVT patients. The usage of AFVCM shunting to prevent postoperative hydrocephalus after FVT resection is not widely practice in neurosurgical centers all over the world except our institution. Thus, we explored the effectiveness of the AFVCM shunting in preventing postoperative hydrocephalus after FVT resection. We also explore the cost and average duration of hospital stay of patients who underwent AFVCM shunting as against patients who did receive AFVCM shunting.

## 2. Materials and methods

### 2.1. Study population

The data of patients who underwent surgery of the fourth ventricle tumor between January 2019 and December 2022 at the Department of Neurosurgery in West China Hospital of Sichuan University were retrospectively retrieved from the hospital records. This study was approved by West China Hospital Research Committee with ethics approval reference 2020-141. Patients, parents to kids as well as their relatives were appropriately informed about our aim to include them in a study and they liberally consented to the use of their documented information. Written informed consents were taken from all the patients as well as parents of children included in the study. Also, written consents for publication were signed by all the patients as well as parents of children included in the study. The hospital also permitted the use of their information for publication. All methods were performed in accordance with the relevant guidelines and regulations.

**Figure 1.**
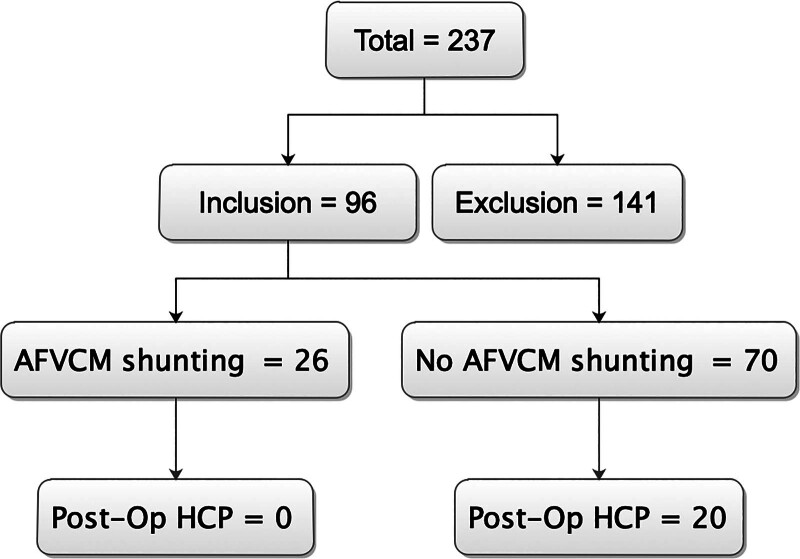
Patients stratifications as per inclusion and exclusion criteria as well as patient’s categorization into AFVCM shunting and no AFVCM shunting groups and development of postoperative hydrocephalus. AFVCM = aqueduct-fourth ventricle-cisterna magna.

A total of 237 fourth ventricle tumor cases were retrospectively retrieved from our hospital records out of which 96 cases had superior tumor extension into the aqueduct and failed total tumor resection as was documented in the surgical notes (Fig. 1). Further criteria for the 96 selective cases were: single intracranial neoplasm detected by preoperative magnetic resonance imaging (MRI); surgical resection of the lesion; a tumor confirmed by pathologic findings. Patients were excluded if there were signs of multiple tumors or repeated surgery for tumor; the patients underwent biopsy rather than resection. Also, we further retrieved 26 cases in whom surgical notes indicated that AFVCM shunting were carried out during surgery out of the 96 cases retrieved from the records. Thus, in 70 patients, no AFVCM shunting were carried out as per the surgical notes’ records (Fig. 1). All tumor resections and AFVCM shunting procedures were carried out by a single senior neurosurgeon. AFVCM shunting was performed in patients with radiological evidence of narrowed cerebral aqueduct or preexisting mild hydrocephalus; intraoperative confirmation of tumor adherence to the fourth ventricular floor, increasing the risk of postoperative CSF obstruction; subtotal tumor resection (100% of AFVCM group vs. 55.7% in non-AFVCM group, *P* < .0001), as residual tumor mass raised concerns about CSF pathway obstruction. Also, “patient selection” criteria excluded individuals with severe intraventricular hemorrhage (CSF erythrocyte count > 10,000/μL) or high infection susceptibility. It is worth noting that AFVCM shunting was intended as a temporary measure (mean duration: 7 days), reducing long-term blockage risk.

## 3. Preoperative radiology and surgical procedures

**Figure 2.**
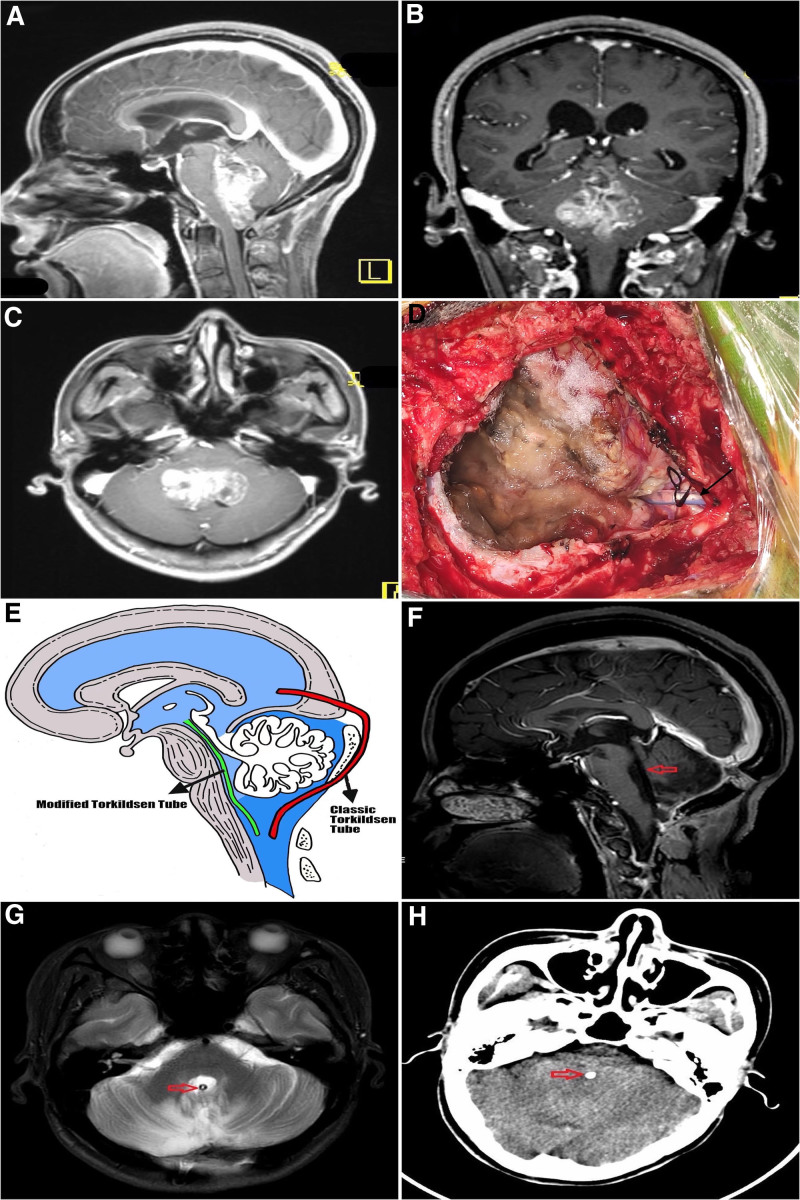
Are pre-, intra- and postoperative radiological and surgical images one of the patients who received AFVCM shunting. (A) Is preoperative sagittal MRI showing fourth ventricular tumor. (B) Is preoperative coronal MRI showing forth ventricular tumor. (C) Is preoperative axial MRI showing forth ventricular tumor. (D) Is an intraoperative image showing the AFVCM shunt in situ with a non-absorbable suture fixing the catheter onto the dura on the foramen magna (black arrow = catheter + suture). (E) Is sketched image showing the precise location of the AFVCM shunt/tube as against the classic Torkildsen shunt/tube. (F) Is postoperative sagittal MRI showing the fourth ventricular after tumor resection (red arrow = the fourth ventricle). (G) Is postoperative axial MRI showing the AFVCM shut in situ after tumor resection (red arrow = AFVCM shunt). (H) Is postoperative CT scan showing the modified Torkildsen shut in situ after tumor resection (red arrow = AFVCM shunt). AFVCM = aqueduct-fourth ventricle-cisterna magna, MRI = magnetic resonance imaging.

In all patients, preoperative MRI was used to establish FVTs which extended into the aqueduct (Fig. 2A–C). The surgical procedures were carried out as previously described.^[4]^ After general anesthesia, resections of the tumors were performed with the patients in the park bench position and their heads fixed in Mayfield 3 keys’ head support system. In all patients, routine electromyographic as well as auditory brainstem responses were utilized to monitor the cranial nerves according to our hospital protocols. The lesions were approached through a standard midline suboccipital approach and a senior neurosurgeon with over 30 years’ experience in the resection of posterior fossa lesions carried out the tumor resections and the AFVCM shunting procedures. Tumor resection method was a piece meal resection approach until a maximum tumor resection was achieved or total resection of tumor depending on the size and tumor type involved. Ventricular catheters were inserted from the orifices of aqueducts through fourth ventricles towards the cisterna magna in the AFVCM shunting group after the tumors were resected (Fig. 2D and E). The lower ends of the catheters were fixed onto the duras on the foramen magnums by non-absorbable sutures. The bone flaps were replaced and the skin closed accordingly. Immediate postoperative managements were carried out as previously described.^[4]^ The key postoperative complications which are relevant to our study, and thus monitored after our procures were postoperative hydrocephalus and intracranial infections. Rigorous intraoperative irrigation wes carried out to minimize erythrocyte load in the CSF.

## 4. Postoperative radiology and hydrocephalus assessment

In all patients both postoperative MRI (Fig. 2F and G) and CT (Fig. 2H) was done after the surgeries. The extent of resection was evaluated by reviewing the postoperative MRI images obtained within 72 hours after surgical treatment, and the gross-total resection was defined as complete tumor resection with no evidence of residual tumor on postoperative MRI. Assessment of hydrocephalus was made by an Evans index (the ratio of the maximal width of the frontal horns to the maximum inner skull diameter) larger than 0.3 with or without clinical symptoms and signs such as headache, nausea, vomiting, lethargy, and papilledema. Intracranial infections were initially established via body temperature increase above 37°C and confirmed by CT and MRI. The duration of hospital stays and the total cost of treatment of all the patients were retrieved from the patients’ records. All patients where follow-up closely on outpatients bases with the aim of detecting any long-term complications using MRI and CT.

## 5. Statistical analysis

Data analysis was performed using the SPSS software version 23.0 (IBM Corp., Armonk, New York, USA). The results of Fisher exact test are reported for two-by-two tables; ordinal variables, and the unpaired *t*-test or analysis of variance was used for continuous variables. The chi-square test as well as the Fisher exact test were employed to complete the univariate analysis of patients with and without postoperative hydrocephalus. The Mann–Whitney test was used to assess differences in continuous variables between shunt group and no shunt group. The significant factors in the univariate analysis were used as covariates in the multivariate analysis, which was performed using logistic regression. All statistical tests were 2-sided; if *P* < .05, the data were considered statistically significant.

## 6. Results

### 6.1. Patients’ characteristics

In all, a total of 96 patients were included in the study after applying our inclusion and exclusion criteria above (Fig. 1). Our retrospective data retrieved revealed that AFVCM shunting was carried out in 26 cases while no shunting was carried out in 70 patients out of the 96 selective cases. The patients included 52 males and 44 females. Also, in the AFVCM shunting group, out of the 26 patients, 14 were males while 12 were females. In the no AFVCM shunting group, out of the 70 patients, 38 were males while 32 were females. No significant statistical difference between the sexes (*P* = .2952) was observed in this study with univariate analysis (Table 1).

**Table 1:** Patients’ characteristics of all the included patients.

Patients’ characteristics	AFVCM shunting (N = 26)	No AFVCM shunting (N = 70)	*P*-value
Sex			
Male	14	38	.2952*
Female	12	32	
Age			
<5	3	6	.8338*
5–18	6	14	
>18	17	50	
Tumor size (mm)	37 ± 5	40 ± 4	.2356*
Subtotal tumor resection	26	39	<.0001‡
Tumor pathology			
Ependymoma	16	39	
Medulloblastoma	4	12	
Astrocytoma	2	7	
Hemangioblastoma	1	4	
Cholesteatoma	1	3	
Choroid plexus papilloma	1	2	
Metastatic	0	1	
Others	1	2	

*P* < .05 is considered statistically significant.

AFVCM = aqueduct-fourth ventricle-cisterna magna.

* Chi-square tests.

† Mann–Whitney test.

‡ Fisher exact test.

According to age stratification, out of the 96 patients included in the study, 9 patients were < 5 years old, 20 patients were between 5 and 18 years while 67 patients were > 18 years old. Age < 5 years is considered the preschool age. Age 5 and 18 is considered schooling age while age > 18 is considered adults. In the AFVCM shunting group, 3 patients were < 5 years old, 6 patients were between 5 and 18 years while 17 patients were > 18 years old. In the no AFVCM shunting group 6 patients were < 5 years old, 14 patients were between 5 and 18 years while 50 patients were > 18 years old. No significant statistical difference between the age’s groups (*P* = .8338) was observed in this study with univariate analysis (Table 1).

## 7. Preoperative radiology and surgical outcome

In all 96 patients, preoperative MRI established FVTs which extended into the aqueduct (Fig. 2A–C). Also, the ventricular catheters were inserted from the orifice of aqueducts through fourth ventricles towards the cisterna magna in the AFVCM shunting group after the tumors were removed (Fig. 2D and E). The tumor sizes in the AFVCM shunting group ranged from 35 ± 5 while the tumor sizes in the no AFVCM shunting group ranged from 40 ± 4. In all, 12 patients in the AFVCM shunting group had preoperative hydrocephalus while 20 patients had preoperative hydrocephalus in the no AFVCM shunting group (Table 1). However, is no statistical significance difference between the 2 groups (*P* = .1051) with univariate analysis. Furthermore, there is no statistical significance difference in tumor sizes and the occurrence of postoperative hydrocephalus (*P* = .2356) with univariate analysis.

In all the 26 patients included in the AFVCM shunting group, we achieved subtotal tumor resections which were the bases for the insertions of the AFVCM shunts. Interesting, none of the patients developed postoperative hydrocephalus. On the other hand, we achieved subtotal tumor resection in 39 patients out of the 70 patients included in the no AFVCM shunting group. Furthermore, 20 patients developed postoperative hydrocephalus out of the 39 patients in whom we achieved subtotal tumor resections. There is significant statistical difference between subtotal tumor resections and the development of postoperative hydrocephalus (*P* < .0001) with univariate analysis. All of the 20 patients who developed postoperative hydrocephalus underwent postoperative VP shunts to relieve their hydrocephalus. However, 5 of the patients received emergency bur hole on the lateral ventricles because those patients’ conditions deteriorated rapidly.

Tumor pathological evaluation revealed that out of the 96 patients included in the study, 55 patients had ependymoma, 16 had medulloblastoma, 9 had astrocytoma, 5 had hemangioblastoma, 4 had cholesteatoma, 3 had choroid plexus papilloma, 1 had metastatic tumor and 3 patients were classified into others. The stratifications of the patients according to the AFVCM shunting group and no AFVCM shunting group is as shown in Table 1.

## 8. Postoperative and postradiological Outcomes

In all patients, both postoperative MRI (Fig. 2F and G) and CT (Fig. 2H) were done as per our hospital protocols. In all 20 patients developed postoperative hydrocephalus out of the 96 patients included in this study (Table 2). All the 20 patients who developed postoperative hydrocephalus are in the no AFVCM shunting group. No patient in the AFVCM shunting group developed postoperative hydrocephalus. We observed a significant statistical difference between the AFVCM shunting group and no AFVCM shunting group (*P* = .0062) with univariate analysis. Thus, AFVCM shunting salvaged postoperative hydrocephalus. Also, a total of 3 patients developed postoperative intracranial infection. Remarkably, 2 patients were in the no AFVCM shunting group while 1 patient was in the AFVCM shunting group (*P* = .4651) with univariate analysis.

**Table 2:** Comparison of patient outcomes between AFVCM shunting and no AFVCM shunting group.

	AFVCM shunting (N = 26)	No AFVCM shunting (N = 70)	*P*-value
Postoperative hydrocephalus	0	20	.0062*^,^†
Intracranial infections	1	2	.4651†
Average hospitalization duration (d)	12	14	.3295†
Average medical cost (Yuan)	50,987	79,034	.0166‡

*P* < .05 is considered statistically significant.

AFVCM = aqueduct-fourth ventricle-cisterna magna.

* Chi-square test.

† Fisher exact test.

‡ Mann–Whitney test.

Interestingly, multivariate analysis (Table 3) revealed that type of tumor (*P* = .017; OR = 1.076; 95% CI = 0.814–1.422), tumor size (*P* = .025; OR = 1.057; 95% CI = 0.516–2.165), residual tumor (*P* = .005; OR = 1.466; 95% CI = 0.833–2.581) were factors associated with postoperative hydrocephalus. However, AFVCM shunting (*P* = .029; OR = 5.47265E−13; 95% CI = 0.000–inf) salvaged postoperative hydrocephalus. Also, postoperative infection was not associated with postoperative hydrocephalus with multivariate analysis (*P* = .057; OR = 2.326; 95% CI = 0.126–42.875). Furthermore, the average duration of hospital stays in the AFVCM shunting group was 12 days while the average duration of hospital stays in the no AFVCM shunting group was 14 days (*P* = .3295) with univariate analysis. Very interesting, the average cost of hospital stays in the AFVCM shunting group was CN ¥ 50,987, while the average cost of hospital stay in the no AFVCM shunting was CN ¥ 76, 034 (*P* = .0166) with univariate analysis. Thus, there is significant statistical difference between the average cost of hospital stay in the AFVCM shunting group and no AFVCM shunting group (Table 2).

**Table 3:** Multivariate analysis of factors associated with postoperative hydrocephalus.

	OR	95% CI	*P*-value
Sex	0.819126328	0.255–2.636	.737948
Age	1.149781069	0.602–2.196	.672384
Type of tumor	1.075624833	0.814–1.422	.017391
Tumor size (mm)	1.056601392	0.516–2.165	.025154
Residual tumor	1.466186913	0.833–2.581	.005279
AFVCM shunting	5.47265E−13	0.000–inf	.02857
Postoperative infection	2.326001143	0.126–42.875	.05702

*P* < .05 is considered statistically significant.

CI = confidence interval, OR = odds ratio.

## 9. Follow-up of patients

The follow-up period for the AFVCM shunting group was between 9 and 36 months with a mean follow-up of 24.1 months while the follow-up period for the no AFVCM shunting group was between 8 and 34 months with a mean follow-up of 20.7 months. We did not observe any scarring in the AFVCM shunting group on MRI as well as CT and all the shunts are still patent and functioning well as per postoperative Evans index assessments with no complications and no reoperations. All patients are still being followed very closely in outpatient departments. Also, we did not observe any mortality and no patients are lost on follow-up.

## 10. Discussion

The FVT is typically depicted with high rate of postoperative hydrocephalus due to the compression of tumor on the fourth ventricle.^[2–4]^ In our current cohort, AFVCM shunting may be a prophylactic procedure for postoperative hydrocephalus in patients with superior tumor extension into the aqueduct and failed total tumor resection during FVT surgeries. Anatomically, the roof as well as walls of the fourth ventricle contain vital structures of the efferent cerebellar pathway such as the dentate nuclei as well as superior cerebellar peduncle while the floor of the fourth ventricle, formed by the brainstem, contains numerous cranial nerve nuclei on its surface.^[8,9]^ Our team earlier on identified superior tumor extension into the aqueduct as significant risk factors for the development of postoperative hydrocephalus.^[4]^

Also, stenosis as a result of inflammatory process and scarring involving the ependymal lining at the cerebral aqueduct as well as CSF pathways such as the ventricles have been observed after surgeries and infections around these CSF pathways.^[5–7,29]^ These occurrences have described as entrapped, trapped, ballooned fourth ventricle, and double compartment hydrocephalus.^[5,30]^ Interestingly, our multivariate analysis revealed that type of tumor, tumor size, residual tumor were factors associated with postoperative hydrocephalus while AFVCM shunting prevented postoperative hydrocephalus. Notably, postoperative infection was not associated with postoperative hydrocephalus with multivariate analysis.

The findings were suggestive that the features above may be used to preoperatively identify patients at high risk of postoperative hydrocephalus after resection of the FVT. Based on these findings, our current study exhibits AFVCM shunting as prophylactic measure for postoperative hydrocephalus after FVT removal by placing a catheter from the aqueducts through fourth ventricles towards the cisterna magna. Current utilized treatment modalities such as VP shunting and ETV for postoperative hydrocephalus are both associated with failures. Notably, our AFVCM shunting is for prophylactic purposes and not curative procedure like the VP shunting and ETV shunting.

Our current cohort revealed an association between subtotal tumor resection and the development of postoperative hydrocephalus. However, the size of the tumor resected did not influence the occurrence of postoperative hydrocephalus. Pathological evaluation of tumor samples revealed that most of our patients had ependymoma, followed by medulloblastoma, astrocytoma, hemangioblastoma, cholesteatoma, choroid plexus papilloma, metastatic tumor and others in a descending order of frequency. However, the type of tumor did not have any influence on the development of postoperative hydrocephalus following tumor resection.

Earlies cohorts demonstrated that younger patients with posterior fossa lesions are at higher risk of developing persistent hydrocephalus after tumor resection.^[11,31–34]^ Precisely some cohort revealed that age < 3 years at posterior fossa tumor surgery is a considerable predictor of postoperative CSF diversion.^[31,33]^ These studies predicated that younger patients with FVTs are often associated with higher incidence of congenital as well as malignant tumors which are normally accompanied by leptomeningeal metastases resulting in the impairment of CSF absorption at the subarachnoid level.^[34–36]^

In our current cohort, although our patient categories comprised of both children and adults, we did not find an association between age and the development of postoperative hydrocephalus. Also, the sex of the patient did not influence the occurrence of postoperative hydrocephalus following tumor resections. Our earlier cohort demonstrated that superior extension of posterior fossa tumor was a significant predictor for postoperative hydrocephalus and the bases for postoperative CSF division, while other extensions did not pose risks factors necessitating postoperative shunting.^[4]^

It was speculated that inflammation after resection of tumors might lead in stenosis of the CSF pathways in patients with tumor extension into the aqueduct, foramina of Luschka, as well as foramen of Magendie necessitating CSF division.^[4]^ It was also observed that FVT resection often developed adhesive arachnoiditis as well as secondary hydrocephalus necessitating CSF division. Furthermore, factors like the presence of postoperative cerebellar edema contributed to postoperative hydrocephalus necessitating CSF division. In our current cohort, we detected that subtotal tumor resection contributes to postoperative edema of surrounding structures resulting in the stenosis of the CSF pathways leading to postoperative hydrocephalus. Also, the residual tumor after resection triggered adhesive arachnoiditis leading to tissue edema and hydrocephalus.

The sporadic requirement of postoperative CSF shunting following FVT resection has been reported in the literature.^[37]^ An earlier cohort reported a postoperative shunting rate of 30% following FVT resections in pediatric patients.^[38]^ Also, a similar cohort with 26 patients with fourth ventricle ependymomas who underwent surgery reported a shunting rate of 23%.^[39]^ Furthermore, another cohort report an incidence rate of 22% postoperative hydrocephalus who required CSF diversion following tumor resection with majority of the patients being children.^[37]^

ETV is another widely use treatment modalities for the management of hydrocephalus in patients with FVTs.^[19–23]^ ETV appear to be more safe and durable means of controlling obstruction hydrocephalus compared to shunting.^[19,20,22]^ Although both modalities are associated with failures, the risk of ETV failure may be lower than the risk of shunt failure surgery.^[19,20,22]^ Nevertheless, patients who develop communication hydrocephalus after FVT tumor resection could benefit from shunt rather than ETV.^[4,22]^ Also, the presence of postoperative cerebellar edema especially in cases with triventricular hydrocephalus seen on imaging, favored ETV rather than shunt insertion.^[4,22]^

In our current cohort, AFVCM shunting in patients with concurrent superior tumor extension into the aqueduct and failed total resection of the tumors prevented patients from developing postoperative hydrocephalus following tumor resections. Our AFVCM shunting although similar to Torkildsen shunt because in both procedures CSF drainage if confined inside the skull is different because the location of shunts in our study were AFVCM (Fig. 2E). The original Torkildsen shunting was a novel remedy to the challenges of treating noncommunicating hydrocephalus.^[25]^ In the original procedure, a bur hole was positioned for a ventriculography via one of the occipital horns of the lateral ventricle followed by a suboccipital craniotomy and a connection of a rubber tube between the occipital horn and the cisterna magna.^[25]^

Subsequently, a Nélaton catheter was placed extracranially underneath the scalp.^[25]^ Thus, the Torkildsen shunting is a simple tubing between 2 CSF spaces to bridge them. The original procedure was done under local anesthesia and both ventricular puncture as well as suboccipital decompressive evaluation were utilized on a routine basis at that time, so this procedure was a natural step.^[25]^ The surgical procedure for the Torkildsen shunt although very simple, can be technically more challenging than VP shunt.^[26]^ It does not necessitate any extraordinary shunt valve to regulate draining pressure.

Also, although the patency of the shunt cannot be determined, mild to moderately reduction in ventricular size within a few weeks and resolution of initial clinical signs and symptoms after the surgery sturdily support the modified Torkildsen shunt plays a fundamental role in improving hydrocephalus. Furthermore, the modified Torkildsen shunt permits drainage of CSF via a physiologic pathway, thus preventing the complications of shunting CSF into the peritoneal cavity. Moreover, the distance between head and neck is relatively shorter compare to the distance between head and abdomen.^[26]^

In cases of FVT resections, AFVCM shunting can be carried in the same operation and thus avoiding a second operations and preventing the complications of ETV or shunting of CSF into the peritoneal cavity which both increases cost to the patient. Prophylactic AFVCM shunting after resection of FVTs can prevent patient from undergoing emergency lateral ventricular bur-holes because patient’s condition will not deteriorated after surgery as compared to patients who are treated the orthodox ways.

In our current cohort, we observed a significant difference between the average cost of hospital stay in patients who underwent the AFVCM shunting as compare to patient who did not receive AFVCM shunting. We are optimistic that AFVCM shunting could reduce the need for additional surgeries as well as their secondary complication, and thus reduce additional cost to the patient. We did not observe any significant difference in postoperative infection rate between patients who underwent the AFVCM shunting and patients who did not receive AFVCM shunting. Pre- and postoperative hydrocephalus often present with signs and symptoms of increased intracranial pressure such as headache, nausea/vomiting, vertigo, unsteady gait, diplopia and papilledema which hinders the patient’s quality life as well as result in a lengthy hospital stay.^[4,15,16]^

Clinical symptoms and signs such as headache, nausea, vomiting, lethargy and papilledema did not influence our diagnosis of hydrocephalus but rather the Evans’ index. Evans’ index is the ratio of the transverse diameter of the anterior horns of the lateral ventricles to the greatest internal diameter of the skull.^[40,41]^ William Evans in 1942 first described this method of measurement of ventricular size in pediatric patients using pneumoencephalograms.^[40,41]^ This method was later adapted for use on CT scans.^[40,42]^ We utilized this method in both CT and MRI to accurately determine postoperative hydrocephalus according to the variation in ventricular sizes pre- and postoperatively. The follow-up period for the modified Torkildsen group was between 9 and 36 months with a mean follow-up of 24.1 months. We did not observe any scarring and all the shunts are still patent and functioning well with no complications and no reoperations in the AFVCM shunting group.

The high patency in AFVCM shunting may be linked to 3 synergistic factors. First, ‌technical refinement‌ such as rigorous intraoperative irrigation protocols (e.g., warm saline flushing of the aqueduct and shunt catheter) effectively reduces erythrocyte debris and fibrin deposition, minimizing mechanical obstruction risks. Second, ‌patient selection‌ criteria excluded individuals with severe intraventricular hemorrhage (CSF erythrocyte count > 10,000/μL) or high infection susceptibility, thereby avoiding scenarios prone to early shunt failure. Third, ‌short-term application‌ (mean duration: 7 days) limits exposure to chronic complications like biofilm formation or positional catheter migration, as AFVCM is designed to address transient postoperative CSF dynamics rather than chronic hydrocephalus. This targeted approach aligns with emerging strategies in temporary CSF diversion for acute intracranial pressure management.

The avoidance of EVT/VP shunts in this study was driven by 2 primary considerations. First, “anatomical suitability” played a critical role: postresection alterations to the fourth ventricular anatomy, such as distorted landmarks or residual tumor adhesion, often rendered VP shunt placement unsafe due to risks of catheter displacement or cerebellar injury. Second, the “study’s prophylactic aim” prioritized transient CSF diversion for managing acute postoperative edema rather than addressing chronic hydrocephalus, as AFVCM shunts were designed for short-term use (mean: 7 days) to mitigate temporary CSF flow disruption. EVT/VP shunts were reserved for confirmed chronic cases, with 12 non-AFVCM patients requiring permanent diversion after demonstrating persistent hydrocephalus in follow-up imaging. This approach aligns with targeted surgical strategies that balance procedural safety with dynamic postoperative pathophysiology.

Our study has limitation because it is a single-center retrospective type and does not have the rigor of the prospective study. Also, the surgical techniques as well as perioperative management of posterior fossa tumor may vary among hospitals. However, we do not have any bias in this current analysis although multicenter prospective studies with larger samples maybe more conclusive. Also, the follow-up duration in study was relatively shorter. Furthermore, we did not compare the AFVCM shunting and ETV or VP-shunting as preventive procedures for postoperative hydrocephalus since our institution did not have such data. Nevertheless, comparison between ETV, VP-shunting and AFVCM shunting will be explored as prospective study in the future.

Furthermore, we acknowledge the limited pediatric representation in this cohort (n = 9, <18 years). While AFVCM shunting holds theoretical promise for avoiding lifelong VP shunt dependence in children, its underutilization reflects systemic challenges inherent to our institutional context: ‌referral bias‌, as our center primarily manages adult FVT (~80% of referrals), and ‌ethical constraints‌, with parental reluctance toward novel prophylactic interventions despite rigorous consent protocols. This demographic skew underscores the necessity for multicenter pediatric trials to validate AFVCM’s role in younger populations, where avoiding permanent shunts may confer significant developmental and quality-of-life advantages.

## 11. Conclusion

There may be an association between subtotal tumor resection or residual tumor and the development of postoperative hydrocephalus. AFVCM shunting in patients with concurrent superior tumor extension into the aqueduct and failed total resection of the tumors may prevent patients from developing postoperative hydrocephalus following tumor resections. Also, AFVCM shunting may be carried in the same operation thereby reducing cost to the patient. These dual advantages such as hydrocephalus prophylaxis and cost efficiency warrant validation through “multicenter pediatric trials”, as children with FVT such as medulloblastoma often present with aqueductal invasion and face lifelong consequences of VP shunts.

## Author contributions

**Conceptualization:** Min Tang, Seidu A. Richard, Yanming Ren, Bowen Huang, Zhigang Lan, Yuekang Zhang.

**Data curation:** Min Tang, Seidu A. Richard, Yanming Ren, Bowen Huang, Zhigang Lan, Yuekang Zhang.

**Formal analysis:** Min Tang, Seidu A. Richard, Yanming Ren, Bowen Huang, Zhigang Lan, Yuekang Zhang.

**Funding acquisition:** Zhigang Lan.

**Investigation:** Min Tang, Seidu A. Richard, Yanming Ren, Bowen Huang, Zhigang Lan, Yuekang Zhang.

**Methodology:** Min Tang, Seidu A. Richard, Yanming Ren, Bowen Huang, Zhigang Lan, Yuekang Zhang.

**Resources:** Zhigang Lan.

**Supervision:** Zhigang Lan, Yuekang Zhang.

**Validation:** Zhigang Lan, Yuekang Zhang.

**Writing – original draft:** Seidu A. Richard.

**Writing – review & editing:** Min Tang, Seidu A. Richard, Yanming Ren, Bowen Huang, Zhigang Lan, Yuekang Zhang.
